# Modules for the Technical Skills Section of the OSCE Component of the American Board of Anesthesiology APPLIED Examination

**DOI:** 10.15766/mep_2374-8265.10820

**Published:** 2019-04-29

**Authors:** Lauryn R. Rochlen, Vijay Tarnal, Jennifer L. Vance, Erik Alderink, Wendy K. Bernstein

**Affiliations:** 1 Clinical Associate Professor, Department of Anesthesiology, University of Michigan Medical School; 2 Clinical Assistant Professor, Department of Anesthesiology, University of Michigan Medical School; 3 Technology Manager for Simulation and Experiential Learning, Office of Medical Student Education, University of Michigan Medical School; 4 Professor, Department of Anesthesiology and Perioperative Medicine, University of Rochester Medical Center; 5 Vice Chairman of Education, Department of Anesthesiology and Perioperative Medicine, University of Rochester Medical Center

**Keywords:** OSCE, Simulation, Clinical Skills Assessment, American Board of Anesthesiology Certification

## Abstract

**Introduction:**

To assess communication and professionalism, as well as technical skills related to patient care, the American Board of Anesthesiology (ABA) has begun administering an Objective Structured Clinical Examination (OSCE) portion of the APPLIED Examination in addition to the Standard Oral Examination component.

**Methods:**

We created video modules and a curriculum for anesthesiology resident OSCE preparation for the Interpretation of Monitors and Interpretation of Echocardiography components. The modules can be used individually by trainees or included as part of an OSCE workshop led by faculty educators with seven individual stations matching the content of the actual ABA examination. These modules are recommended for all levels of anesthesiology trainees so that they can gain exposure to the format and the fast pace of the examination.

**Results:**

Sixty-six junior and senior anesthesiology residents, fellows, and junior faculty successfully participated in these modules. Seventy-three percent of the participants agreed that after completing these modules, they now had a good understanding of the Interpretation of Monitors and Interpretation of Echocardiography technical skills stations. More than 90% of participants reported that the modules were useful, and more than 70% reported that they now felt prepared for these stations of the OSCE.

**Discussion:**

Developing technical skills stations for deliberate practice and preparation for the ABA OSCE is resource intensive. Finding time and faculty to facilitate OSCE preparation is also challenging. With the video modules and scripts included in this publication, residents can practice independently or as part of larger preparation course.

## Educational Objectives

By the end of this activity, learners will be able to:

1. Describe the content, timing, and structure of the Interpretation of Monitors (IOM) component of the American Board of Anesthesiology (ABA) Objective Structured Clinical Examination (OSCE).
2. Describe the content, timing, and structure of the Interpretation of Echocardiography (IOE) component of the ABA OSCE.
3. Integrate information presented in the IOM module required to identify the correct clinical conditions represented in the IOM module.
4. Identify the view and relevant anatomy and make diagnostic assessments and treatment recommendations for the scenarios represented in the IOE module.
5. Demonstrate proficiency in anesthesiology milestones mapped to these modules.

## Introduction

In March 2018, the American Board of Anesthesiology (ABA) began administering an Objective Structured Clinical Examination (OSCE) portion of the APPLIED Examination in addition to the Standard Oral Examination component. The OSCE was added to the current examination to assess two domains that are perceived as difficult to evaluate in a written or oral examination format, namely, communication and professionalism, as well as technical skills that relate to patient care. According to the OSCE content outline developed by the ABA, examinees participate in a seven-station circuit that evaluates their proficiency in at least seven of the nine following skills.^1^

• Communication and professionalism:
  ○ Informed consent.
  ○ Treatment options.
  ○ Periprocedural complications.
  ○ Ethical issues.
  ○ Communication with other professionals.
  ○ Practice-based learning and improvement.
• Technical skills:
  ○ Interpretation of monitors (IOM).
  ○ Interpretation of echocardiograms (IOE).
  ○ Application of ultrasonography.

Since this is a new assessment and the format has only recently begun being formally tested on any residency trainees, there is a lot of trepidation regarding how to best prepare residents for what they will encounter during such a critical examination.^2^ Residency programs across the country are working to develop educational workshops, seminars, and modules to best ensure success for their anesthesia trainees on the OSCE component.^2^ While many of the professional and communications scenarios are relatively easy to reproduce and encompass common situations and interactions, the skills-based stations may be the hardest to reproduce and test.

Due to the novelty of the OSCE component and minimal availability of training modules, we created an OSCE preparation course for our trainees. Since we could not find any videos similar enough to the examples of the IOM and IOE sections as demonstrated on the ABA website, we created our own. In line with the examples on the ABA website, we created the video modules included in this resource. We have used these as part of a larger preparation course that also includes some of the professionalism and communications sections referenced above.

In this resource, we provide descriptions of the content and structure of the IOM and IOE sections of the exam. The information featured in the resource is used to introduce trainees to what is going to be included and assessed during these two sections of the examination. In order to assist with preparation for this high-stakes examination, we provide video modules that we created for each of these sections. Also included are the accompanying response sheets in line with what will be asked by the ABA during the examination. These modules can be used in a group setting or by individual trainees who are interested in independent practice. They can also be used on their own for a review of the technical skills stations or as part of a larger OSCE preparation course. For training programs that are also interested in using the modules to assist with milestones assessment, we have included potential milestones that are addressed during each module.^3–6^

Anesthesiology residents gain clinical experience with monitor and echocardiographic interpretation during their training. Anesthetic monitors collect physiologic data that can be easily displayed and adapted to reflect changes in homeostasis, thereby allowing prompt recognition of adverse changes, responses to therapeutic interventions, and proper functioning of anesthetic equipment. Monitoring standards set forth by the American Society of Anesthesiologists Committee on Standards and Practice Parameters have been adopted across the specialty for conduct of all general anesthetics, regional anesthetics, and monitored anesthesia care.^7^ Live analysis and alerts in real time may allow for early recognition of adverse events, and therapeutic interventions for such events can improve patient care.^8^

Transesophageal echocardiography (TEE) has become an increasingly important imaging modality for anesthesiologists in the perioperative period for the purpose of identifying and monitoring major cardiac pathophysiology and to help diagnose or rule out causes of hemodynamic instability. TEE has expanded from the cardiac operating room to use in liver transplant cases, major aortic vascular cases, and cases involving patients with cardiac comorbidities. Due to the expanded perioperative use of TEE, the ability to obtain and interpret findings for 11 basic TEE views is now deemed an important skill for anesthesia trainees to gain competency in during their residency training.^9^

The full content outline for the IOE and IOM sections is available on the ABA website (www.theaba.org).^1^ Portions of the APPLIED Examination OSCE content outline used in the resource are reprinted with permission of the ABA. The content outline from the ABA for the IOM station is as follows:

• Relevant parameters that may be included:
  ○ Electrocardiogram.
  ○ Arterial blood pressure: noninvasive (value) or invasive (waveform and value).
  ○ Central venous pressure: waveform and value.
  ○ Pulmonary arterial pressure: waveform and value.
  ○ Pulmonary artery occlusion pressure: value.
  ○ Cardiac output: value.
  ○ Mixed venous oxygen saturation: value.
  ○ Pulse oximetry: waveform and value.
  ○ Capnography: waveform and end-tidal value.
  ○ Airway pressure: waveform and peak, positive end-expiratory pressure values.
  ○ Airway flow: waveform.
  ○ Tidal volume: waveform and end-tidal values.
  ○ Respiratory rate, inspiratory and expiratory times.
  ○ Flow-volume loops: waveform.
  ○ Temperature: value.
• Clinical conditions that may be included:
  ○ Perioperative cardiac events.
  ○ Perioperative respiratory events.
  ○ Other perioperative emergencies.
  ○ Ventilatory modes used in normal and critically ill patients.

The ABA content outline for the IOE station is shown below:

• Biventricular function and wall motion abnormalities.
• Presence or absence of an atrial septal defect.
• Volume status assessment: hypovolemia and response to volume therapy.
• Pulmonary emboli.
• Air emboli.
• Basic valvular lesions.
• Pericardial effusions.
• Aortic dissection.

The information included in this resource was used to introduce trainees to this information.^1^ The ABA does not endorse or attest to the current veracity of the items presented. On the ABA website, there are video examples created by the ABA for each of these stations. Our resource serves as a source of additional video examples that residents and training programs can use for preparation. While individual programs may have created modules that they use for resident preparation, we are not aware of any other published modules available for broad use.

## Methods

The clinical content and physiologic data for the scenarios depicted in the IOM were developed by us. The clinical content and TEE images we obtained for the IOE modules were adapted from actual patients with their consent for use. Common clinical scenarios were used in both modules to ensure broad understanding by trainees.

### Implementation

We administered these two modules as part of a larger OSCE preparation course. We offered one course with a more informal format for residents and a more structured course for fellows and junior faculty who were actively preparing to take their APPLIED Examination. Junior faculty included participants who had completed their anesthesiology residency but had not pursued additional fellowship training. As these courses also incorporated the professionalism and communication sections with the use of standardized actors, we held them in our Clinical Simulation Center, which provided enough space to set up all the different stations.

Administration of these modules to the resident cohort was group based. The videos were played on a large screen for all to view simultaneously. The residents were provided with all the necessary information and handouts. The video was played, and the residents wrote down their responses. At the conclusion of each video module, debriefing occurred as a group. This process was repeated with the next module. It did not matter in which order the modules were presented. Each video was approximately 7 minutes in duration, in line with the timing set by the ABA for each OSCE station. It took approximately 45 minutes to go through the introduction and each module in this group setting. Our group size for this format was five to six residents per course. In this setup, only one facilitator was required.

For the more formal fellow and junior faculty course, the modules were set up as separate stations that the trainees encountered independently. Similar to the actual administration of the ABA OSCE component, the trainees discussed their answers with a faculty facilitator present in the station with them. Debriefing was delayed until each trainee completed each station in the course. This format required one facilitator to be at each of the IOM and IOE stations.

### Interpretation of Monitors Module

*Brief explanation of the IOM station:* In this station, the candidates were presented with three separate scenarios and asked to interpret data from a physiologic display. The video is included as Appendix A. Each scenario began with a short scenario description. A recording of a simulated physiologic monitor was shown to the candidates. Over 60 seconds, the physiologic data shown on the monitor changed from a baseline state to a new, altered state based on an unknown event occurring with the patient. After completion of the evolution in physiologic data, the candidates had 60 seconds to answer two questions, one on the most likely diagnosis that resulted in the changes observed and another regarding features on the monitor that supported the diagnosis. The candidates had to be specific and concise when providing their responses. At the bottom right corner of the screen, the recording of the monitor had a timer that progressed backwards for approximately 60 seconds, apprising the candidates of the remaining time for the scenario. At no point could the candidates advance to the next scenario or go back to the previous one. Information and correct responses for each scenario included in the module are available in the facilitator's guide (Appendix B).

The candidates were provided with the IOM Information for Candidate handout (Appendix C) prior to beginning the module. Candidates used Appendix D to write down their responses.

*How the IOM video module was made*: Physiologic monitoring recordings were created using Laerdal LLEAP software. We captured simulated patient monitor images and sounds using Sony Vegas video-editing software. Texts, timers, and video transitions were created within Sony Vegas, and the final video was created as an MP4 file for compatibility.

*Milestones:* Milestones that could be assessed with the IOM module are Patient Care 4: Management of Peri-Anesthetic Complications and Patient Care 9: Technical Skills: Use and Interpretation of Monitoring and Equipment.^3^

### Interpretation of Echocardiography Module

*Brief explanation of the IOE station:* The IOE station was video based, with intraoperative TEE images obtained by cardiac anesthesiologists board-certified in perioperative TEE. The video is included as Appendix E. The station was divided into three scenarios. For the first part, the candidate had 45 seconds to view the video clip and identify the TEE view and two anatomic structures. For the second part, the candidate had 2 minutes to review a brief history, watch the TEE video, identify the TEE view, and provide the most likely diagnosis based on what the image showed. For the third part, the candidate had 2.5 minutes to identify the TEE image, provide the most likely diagnosis, and discuss how to manage the patient. This part came with a longer case descriptor. At no time could the resident go back to a previous case even if there was extra time at the end of the station. Information and correct responses for each scenario included in the module are available in the facilitator's guide (Appendix B).

The candidates were provided with the IOE Information for Candidate handout (Appendix F) prior to beginning the module. Candidates used Appendix G to write down their responses and Appendix H for a list of the possible views.

*How the IOE video module was made*: The TEE video clips were obtained from actual patients by TEE-certified anesthesiologists at the University of Michigan. The clips were added to a PowerPoint presentation, and then, timing was added and the presentation recorded.

*Milestones:* The milestone that could be assessed by the IOE module is Patient Care 4: Management of Peri-Anesthetic Complications.^3^

### Learner Evaluation

A form for learners to evaluate the modules with was developed using the web-based platform Qualtrics. A link to the evaluation was emailed to each participant following participation in the OSCE preparation course. The evaluatio is included as Appendix I. Questions used a 5-point Likert scale (1 = *Strongly agree*, 5 = *Strongly disagree*). Data were analyzed and summarized using frequencies and percentages. Fisher's exact test was used to test for independence between the respondent's role and response.

## Results

These modules have been used with a total of 66 anesthesiology residents (CA1 = 20, CA2 = 15, CA3 = 13), fellows (14), and junior faculty (four) since January of 2018.

Of the 66 respondents, 73% (48) somewhat agreed or strongly agreed that following participation in this course, they understood the components of the technical skills portion of the ABA OSCE. Ninety-seven percent (64) agreed that the IOM module was useful, while 94% (62) felt the IOE module was useful. Seventy-six percent (50) agreed that they now felt prepared for the IOM station of the APPLIED Examination, and 73% (48) agreed that they now felt prepared for the IOE station of the APPLIED Examination.

Responses did not vary significantly by training level except for the question regarding feeling prepared for the IOE station. Proportionally more senior residents, fellows, and faculty agreed that they felt prepared compared to the junior residents (Figure).

**Figure.**
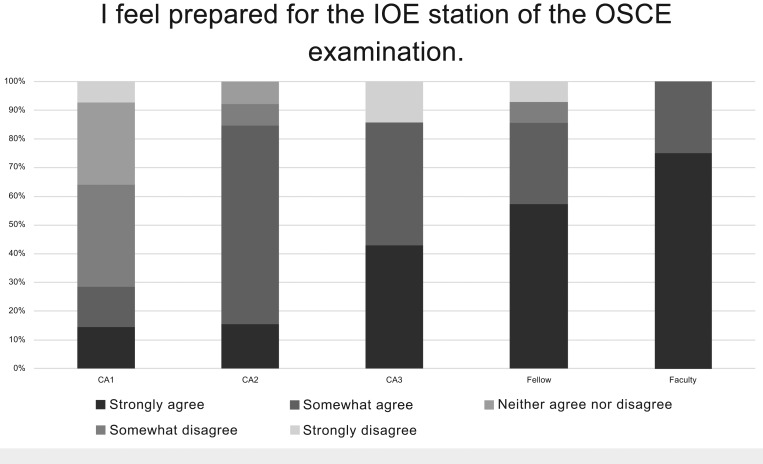
Responses by training level for the Objective Structured Clinical Examination (OSCE) question regarding agreement with feeling prepared for the Interpretation of Echocardiography (IOE) station.

## Discussion

We developed video modules and a curriculum for preparing anesthesiology residents for the IOM and IOE technical skills stations they might encounter during the OSCE component of their APPLIED Examination for board certification in anesthesiology. The curriculum was designed so that the modules could be used individually, together, or as part of a larger OSCE preparation course. The curriculum could also be used for independent study or with a facilitator and multiple participants.

Our primary objective was to develop IOM and IOE video modules that our trainees felt prepared them for the OSCE component. Developing high-fidelity OSCE preparation material can be resource intensive.^2^ Recent surveys of anesthesiology training programs suggest that many programs may not have the number of faculty or financial support needed to be able to provide this type of training for their residents.^4,10^ Preparation for both components of the OSCE is vital as all current residents must now undergo this portion as part of the board certification process. We believe that our instructional videos can encourage and assist other training programs to develop these valuable educational modules. Since we have included potential milestones that relate to the modules, residency programs should benefit from using the modules as part of their milestones assessments.^5,6^

Overall, the participants felt that the modules were helpful and reported that they felt prepared for the technical skills stations following their experience with the curriculum. As of the time of publishing this curriculum, participants had practiced these modules only once. We anticipate that with additional exposure, their confidence level will increase. It is our plan to continue having our trainees practice with this module, as well as to develop new modules.

As part of our results, we saw that the senior participants reported a higher comfort level with the IOE station. This may be due to the fact that many of the junior residents had not had significant exposure to TEE training prior to participating in the OSCE preparation course. However, despite this lack of prior TEE training, they still found practicing the IOE to be helpful overall. Even though anesthesiology trainees do not take this exam until after completion of training, we highly recommend that junior and senior residents participate in OSCE preparation early on in their training to receive the benefits of repeated deliberate practice.

From a practical standpoint, we have found that the video modules are relatively easy to display on the individual computers that examinees use or on a large monitor in a classroom setting. Several residents reported that the quality of the visual aids was excellent. Others commented that the exam felt “very fast paced” and that the audiovisual material was timed too quickly (despite the fact that we followed the actual ABA guidelines). Participants need to be encouraged to pay close attention to the patient information on the first slide. This information may provide specific clues to the diagnoses.

Limitations of the curriculum included scheduling time to get participants relieved from clinical duties in order to participate. This is a common limitation with educational interventions. We held the practice sessions during regularly scheduled resident protected education time. The fellow and junior faculty courses were held in the evening. Another limitation is that each participant had practiced each module only once, which may have resulted in the lower scores regarding feelings of preparation. The evaluation form has been modified to include open-ended questions regarding the strengths and weaknesses of the modules.

## Appendices

A. IOM.mp4
B. Facilitator's Guide.docx
C. IOM Info for Candidate.docx
D. IOM Response Sheet.docx
E. IOE.mp4
F. IOE Info for Candidate.docx
G. IOE Response Sheet.docx
H. List of TEE Views.docx
I. Learner Evaluation.docx All appendices are peer reviewed as integral parts of the Original Publication.
