# Supplementary material for: Modules for the Technical Skills Section of the OSCE Component of the American Board of Anesthesiology APPLIED Examination
Source: MedEdPORTAL. 2019 Apr 29;15:10820. doi: 10.15766/mep_2374-8265.10820 (PMC6507923; doi:10.15766/mep_2374-8265.10820)
Supplement: Supplementary file 1 — A. IOM.mp4 B. Facilitator's Guide.docx C. IOM Info for Candidate.docx D. IOM Response Sheet.docx E. IOE.mp4 F. IOE Info for Candidate.docx G. IOE Response Sheet.docx H. List of TEE Views.docx I. Learner Evaluation.docx [file mep-15-10820-s001.zip › B. Facilitator's Guide.docx]

Facilitator’s Guide

If these modules will be used in a group setting with each resident completing the accompanying response sheet, it is suggested that feedback regarding the residents’ responses during these modules be held until the completion of all modules. If these modules are used on an individual basis with residents reporting their responses directly to a faculty member, it is suggested that feedback regarding responses come at the conclusion of the module.

Residents are not allowed to ask questions of the facilitators during the video modules. They are not allowed to pause the module or go back to an earlier scenario. If a resident does ask a question or request to pause, the facilitator should state that this is not permitted and should remain silent.

For debriefing, it is recommended to go back over the video modules. Each scenario can be discussed with the residents as a group or individually. The ABA is interested in the most likely diagnosis, not a differential. This should be enforced during the debriefing.

*Guide to each scenario in the IOM video*

All scenarios included in this module begin with a brief description of the patient and procedure. There is a countdown from 20 seconds displayed on the bottom corner. The module then transitions to show baseline physiologic monitoring recording. Monitors include: ECG Lead II & V, Pulse Oximetry SpO_2_, Invasive blood pressure, Non-Invasive Blood Pressure, End Tidal CO_2_, Temperature, Fraction of inspired oxygen and End-Tidal Sevoflurane.

The first scenario displays the physiologic parameters of a patient presenting for a laparoscopic hysterectomy and bilateral salpingo-oophorectomy. Within 2 minutes of surgical incision and immediately following insufflation, there are changes noted in the hemodynamic parameters. The patient develops significant hypotension, with no changes in any other physiologic parameter. The most likely diagnosis is hypotension related to insufflation of the peritoneum with gas. The features supporting this are significant hypotension without associated tachycardia due to pre-existing beta-blockage. There are no new ST segment changes. There are no changes in the peak or plateau airway pressures on the ventilator waveforms. The timing of the hemodynamic changes also makes hypotension related to induction of anesthesia or significant blood loss less likely.

The second scenario displays the physiologic parameters from a T6 quadriplegic patient presenting for a urologic procedure. In this scenario, hemodynamic changes are noted ten minutes into the procedure. The patient develops significant hypertension and bradycardia. The most likely diagnosis in autonomic hyper-reflexia. This diagnosis is consistent with hemodynamic changes seen, the patients underlying comorbid condition of paraplegia and the type of surgical procedure.

The third scenario is of a patient presenting for robotic laparoscopic prostatectomy. Standard baseline physiologic monitoring is used for this case. The first image is of the vital signs monitor immediately following induction of general anesthesia. The second image is of the vital signs monitor 5 minutes after 2g cefazolin IV is administered. The patient has developed hypoxemia, hypotension and an obstructive pattern on the capnography. The most likely diagnosis in this scenario is anaphylaxis due to antibiotic administration given the hemodynamic and respiratory changes.

*Guide to each scenario in the IOE video*

The first part consists of a video image, Midesophageal Long-axis view, which the resident must identify from the list of the 11 Basic TEE Views. There are two structures that the resident must identify: the left atrium (LA) labeled with an arrow, and the aortic valve (AV) which is circled.

The second part consists of a short vignette describing a 78 year old female who presents for colon resection due to colon cancer. The patient also has a history of hypertension for which she takes metoprolol. The patient develops hypotension that is resistant to repeated doses of phenylephrine. A TEE probe is placed. The candidate must first identify the TEE view and describe what is seen in the image, ie the Transgastric Mid-papillary short-axis view. The images demonstrate an underfilled left ventricle with normal function suggestive of a hypovolemic state. Beta blocker usage would account for the low heart rate in the setting of hypovolemia.

The third part consists of a longer case vignette describing a 52-year-old male who has suffered a motor vehicle accident. The trauma team placed an arterial line, central line and pulmonary artery catheter during the initial evaluation. The patient is scheduled for an open reduction internal fixation (ORIF) of an open femur fracture. Shortly after induction of general anesthesia, he becomes hemodynamically unstable. The etiology is not immediately clear despite the presence of the pulmonary artery catheter, so a TEE probe are placed by the anesthesiology provider for diagnostic purposes. Two TEE images are shown. The candidate must first identify the Midesophageal Ascending Aortic short- and long-axis views, and report on the aortic dissection which is present. The candidate should then propose a management plan for the patient which would include: strict blood pressure control and heart rate control (avoid tachycardia), confirm appropriate large bore venous access for potential transfusion, and resuscitation, inform the orthopedic surgeon of the type A dissection and the need for immediate cardiac surgical intervention.
