# Supplementary material for: Modules for the Technical Skills Section of the OSCE Component of the American Board of Anesthesiology APPLIED Examination
Source: MedEdPORTAL. 2019 Apr 29;15:10820. doi: 10.15766/mep_2374-8265.10820 (PMC6507923; doi:10.15766/mep_2374-8265.10820)
Supplement: Supplementary file 1 — A. IOM.mp4 B. Facilitator's Guide.docx C. IOM Info for Candidate.docx D. IOM Response Sheet.docx E. IOE.mp4 F. IOE Info for Candidate.docx G. IOE Response Sheet.docx H. List of TEE Views.docx I. Learner Evaluation.docx [file mep-15-10820-s001.zip › C. IOM Info for Candidate.docx]

In this station, you will be asked to interpret data from a physiologic display. You will be presented with three separate scenarios.

Each scenario will begin with a short case description. A recording of a simulated physiologic monitor will then be shown.

In each scenario, changes will occur in the monitor recording. These changes may occur while the recording is playing, or you will be shown two separate recordings, one before and one after the changes have occurred.

You will be told which format will be used as a part of the case description for each scenario.

After you watch the monitor recording, you will have approximately 60 seconds to answer the following two questions about the scenario:

A. What is the most likely diagnosis that resulted in the changes observed?

B. What features on the monitor support the diagnosis?

Please be specific and concise when providing your responses. A timer at the right bottom corner of the screen will count backwards for approximately 60 seconds to apprise you of the remaining time for the answering portion of each scenario.

Each recording will be played only once; you will NOT have the opportunity to go back and review the recordings.
