# Supplementary material for: Modules for the Technical Skills Section of the OSCE Component of the American Board of Anesthesiology APPLIED Examination
Source: MedEdPORTAL. 2019 Apr 29;15:10820. doi: 10.15766/mep_2374-8265.10820 (PMC6507923; doi:10.15766/mep_2374-8265.10820)
Supplement: Supplementary file 1 — A. IOM.mp4 B. Facilitator's Guide.docx C. IOM Info for Candidate.docx D. IOM Response Sheet.docx E. IOE.mp4 F. IOE Info for Candidate.docx G. IOE Response Sheet.docx H. List of TEE Views.docx I. Learner Evaluation.docx [file mep-15-10820-s001.zip › D. IOM Response Sheet.docx]

**Scenario 1**

A. What is the most likely diagnosis that resulted in the changes observed?

B. What features on the monitor support the diagnosis?

**Scenario 2**

A. What is the most likely diagnosis that resulted in the changes observed?

B. What features on the monitor support the diagnosis?

**Scenario 3**

A. What is the most likely diagnosis that resulted in the changes observed?

B. What features on the monitor support the diagnosis?
