# Supplementary material for: Modules for the Technical Skills Section of the OSCE Component of the American Board of Anesthesiology APPLIED Examination
Source: MedEdPORTAL. 2019 Apr 29;15:10820. doi: 10.15766/mep_2374-8265.10820 (PMC6507923; doi:10.15766/mep_2374-8265.10820)
Supplement: Supplementary file 1 — A. IOM.mp4 B. Facilitator's Guide.docx C. IOM Info for Candidate.docx D. IOM Response Sheet.docx E. IOE.mp4 F. IOE Info for Candidate.docx G. IOE Response Sheet.docx H. List of TEE Views.docx I. Learner Evaluation.docx [file mep-15-10820-s001.zip › F. IOE Info for Candidate.docx]

In this station, you will be presented with 3 separate case scenarios. Each scenario will begin with instructions and a short case description, if relevant. After you watch the echocardiogram recordings, you will be asked questions about the scenario by an examiner. The scenarios will include 1 image identification, 1 short case, and 1 long case as described below.

During this session, you will be scored on the numbered items listed below. The examiner may also ask you to describe the features of the recording that you used to make your decisions for the short and long cases.

Part 1. Image identification – you will view a single echocardiogram loop with labeled structures

Part 2. Short Case – you will view 1 or more echocardiogram loops. If more than 1 loop is presented, assume it is obtained from the same patient.

Part 3. Long case – you will view multiple echocardiograms loops from the same patient.

Each recording will be played only once; you will NOT have the opportunity to go back and review the recordings.

When you finish answering the questions associated with one case scenario, you may request to view the next recording at any time, but you will NOT be able to go back to other scenarios later. Each of the 3 case scenarios is separate and has no connection with the preceding or subsequent scenario. You will provide all answers verbally to an examiner who will be in the room. The examiner will NOT provide additional information about the cases or the images.
