# Supplementary material for: Modules for the Technical Skills Section of the OSCE Component of the American Board of Anesthesiology APPLIED Examination
Source: MedEdPORTAL. 2019 Apr 29;15:10820. doi: 10.15766/mep_2374-8265.10820 (PMC6507923; doi:10.15766/mep_2374-8265.10820)
Supplement: Supplementary file 1 — A. IOM.mp4 B. Facilitator's Guide.docx C. IOM Info for Candidate.docx D. IOM Response Sheet.docx E. IOE.mp4 F. IOE Info for Candidate.docx G. IOE Response Sheet.docx H. List of TEE Views.docx I. Learner Evaluation.docx [file mep-15-10820-s001.zip › G. IOE Response Sheet.docx]

**Part 1. Image identification**

A. Select the proper name of the view from the list of standard views

B. Identify each of the labeled structures

Red arrow =

Red circle =

**Part 2. Short Case**

A. Select the proper name of the view from the list of standard views

B. Provide the most likely diagnosis based on the echocardiographic findings

**Part 3. Long case**

A. Select the proper name of the view from the list of standard views

B. Provide the most likely diagnosis based on the echocardiographic findings

C. Explain how you would manage this patient and why
