# Supplementary material for: Modules for the Technical Skills Section of the OSCE Component of the American Board of Anesthesiology APPLIED Examination
Source: MedEdPORTAL. 2019 Apr 29;15:10820. doi: 10.15766/mep_2374-8265.10820 (PMC6507923; doi:10.15766/mep_2374-8265.10820)
Supplement: Supplementary file 1 — A. IOM.mp4 B. Facilitator's Guide.docx C. IOM Info for Candidate.docx D. IOM Response Sheet.docx E. IOE.mp4 F. IOE Info for Candidate.docx G. IOE Response Sheet.docx H. List of TEE Views.docx I. Learner Evaluation.docx [file mep-15-10820-s001.zip › H. List of TEE Views.docx]

List of Echocardiogram Views

a. Midesophageal Four Chamber

b. Midesophageal Two Chamber

c. Midesophageal Long Axis

d. Midesophageal Ascending Aortic Long Axis

e. Midesophageal Ascending Aortic Short Axis

f. Midesophageal Aortic Valve Short Axis

g. Midesophageal Right Ventricular Inflow-Outflow

h. Midesophageal Bicaval

i. Transgastric Midpapillary Short Axis

j. Descending Aortic Short Axis

k. Descending Aortic Long Axis
