# Supplementary material for: Modules for the Technical Skills Section of the OSCE Component of the American Board of Anesthesiology APPLIED Examination
Source: MedEdPORTAL. 2019 Apr 29;15:10820. doi: 10.15766/mep_2374-8265.10820 (PMC6507923; doi:10.15766/mep_2374-8265.10820)
Supplement: Supplementary file 1 — A. IOM.mp4 B. Facilitator's Guide.docx C. IOM Info for Candidate.docx D. IOM Response Sheet.docx E. IOE.mp4 F. IOE Info for Candidate.docx G. IOE Response Sheet.docx H. List of TEE Views.docx I. Learner Evaluation.docx [file mep-15-10820-s001.zip › I. Learner Evaluation.docx]

Learner Evaluation

Please answer these questions regarding the Interpretation of Monitoring (IOM) and Interpretation of Echocardiography(IOE) stations from the OSCE preparation course you participated in.

Q1. After participating in this course, I understand the components of the technical skills portion of the ABA OSCE examination.

- Strongly agree
- Somewhat agree
- Neither agree nor disagree
- Somewhat disagree
- Strongly disagree

Q2. The video module for the Interpretation of Monitors(IOM) station was helpful.

- Strongly agree
- Somewhat agree
- Neither agree nor disagree
- Somewhat disagree
- Strongly disagree

Q3. I feel prepared for the IOM station of the OSCE examination.

- Strongly agree
- Somewhat agree
- Neither agree nor disagree
- Somewhat disagree
- Strongly disagree

Q4. The video module for the Interpretation of Echocardiography station was helpful.

- Strongly agree
- Somewhat agree
- Neither agree nor disagree
- Somewhat disagree
- Strongly disagree

Q5. I feel prepared for the IOE station of the OSCE examination.

- Strongly agree
- Somewhat agree
- Neither agree nor disagree
- Somewhat disagree
- Strongly disagree

Q6. What is your current role?

- CA1
- CA2
- CA3
- Fellow
- Faculty
